# Hif-1α Inhibitors Could Successfully Inhibit the Progression of Differentiated Thyroid Cancer in Vitro

**DOI:** 10.3390/ph13090208

**Published:** 2020-08-24

**Authors:** Min-Hee Kim, Tae Hyeong Lee, Jin Soo Lee, Dong-Jun Lim, Peter Chang-Whan Lee

**Affiliations:** 1 Division of Endocrinology and Metabolism, Department of Internal Medicine, Eunpyeong St. Mary’s Hospital, College of Medicine, The Catholic University of Korea, Seoul 03312, Korea; benedict@catholic.ac.kr (M.-H.K.); jinsoo21@catholic.ac.kr (J.S.L.); 2 Department of Biomedical Sciences, Asan Medical Center, University of Ulsan College of Medicine, Seoul 05505, Korea; T.Hyeong.Lee@gmail.com; 3 Division of Endocrinology and Metabolism, Department of Internal Medicine, Seoul St. Mary’s Hospital, College of Medicine, The Catholic University of Korea, Seoul 06591, Korea

**Keywords:** HIF-1α, IDF-11774, thyroid cancer

## Abstract

Hypoxia-inducible factor (HIF)-1α plays an important role in cancer progression. In various cancers, including thyroid cancer, overexpression of HIF-1α is related to poor prognosis or treatment response. However, few studies have investigated the role of HIF-1α inhibition in thyroid cancer progression. We evaluated the utility of the HIF-1α inhibitor IDF-11774 in vitro utilizing two thyroid cancer cell lines, K1 and BCPAP. Both cell lines were tested to elucidate the effects of IDF-11774 on cell proliferation and migration using soft agar and invasion assays. Here, we found that a reduction of HIF-1α expression in BCPAP cells was observed after treatment with IDF-11774 in a dose-dependent manner. Moreover, cell proliferation, migration, and anchorage-independent growth were effectively inhibited by IDF-11774 in BCPAP cells but not in K1 cells. Additionally, invasion of BCPAP but not K1 cells was controlled with IDF-11774 in a dose-dependent manner. Our findings suggest that promoting the degradation of HIF-1α could be a strategy to manage progression and that HIF-1α inhibitors are potent drugs for thyroid cancer treatment.

## 1. Introduction

Over the past few decades, a steep increase in the incidence of thyroid cancer has been observed. While detection of papillary thyroid microcarcinomas smaller than 1 cm is the main reason for this explosive increase, the incidence of thyroid cancers larger than 1 cm has also increased [1]. In a recent study, increased mortality of thyroid cancers across all subtypes, including differentiated cancer, was observed [2]. As most differentiated thyroid cancers (DTCs) show favorable prognoses, active surveillance of thyroid cancer, which does not harbor risk factors for poor prognosis, could be considered in current clinical practices. However, it is not uncommon to encounter advanced thyroid cancer, including both metastatic differentiated and dedifferentiated (poorly differentiated and anaplastic) cancer cases that do not respond to conventional therapy, such as thyroidectomy and radioactive iodine therapy. Patients with metastatic DTC that do not respond to radioactive iodine treatment have shown less than 10% of 10-year overall survival [3]. Poorly differentiated and anaplastic cancers also show very low 5-year overall survival rates of 50–60% and about 10%, respectively [4,5].

The effective treatment options for advanced thyroid cancers are very limited. In radioactive iodine-responsive differentiated thyroid cancers, surgery and radioactive iodine ablation could effectively control the progression of disease. However, in refractory differentiated, poorly differentiated, or anaplastic thyroid cancers, there is no effective treatment. Conventional chemotherapy has shown disappointing effectiveness, with a response rate of less than 30% [6]. Several tyrosine kinase inhibitors (TKIs), such as sorafenib and lenvatinib, are currently indicated for refractory DTC cases [7]. However, TKIs usually show tumoristatic rather than tumoricidal effects, [8], which can lead to increased risk of flare-ups after TKI discontinuation [9,10]. TKIs may also increase the risk of tumor progression through the tumor escape phenomenon by activating alternative signaling pathways in the tumor microenvironment [11]. In this context, treatments targeting the key molecules involved in the maintenance of the tumor microenvironment, such as hypoxia-inducible factor (HIF), could at least partially overcome the limitations of currently used TKIs [12]. In addition, in our previous study that evaluated factors associated with ^18^F-fluorodeoxyglucose avidity in primary thyroid cancer, HIF-1α was the candidate protein identified for its involvement in altered metabolism and acquisition of aggressiveness [13]. Other studies have also identified associations between HIF-1α and high TNM stage or lymph node metastases in immunohistochemical studies of human thyroid cancer tissues [14,15].

HIF is a transcriptional factor consisting of an oxygen-labile α unit and a constitutively expressed β unit. It is involved in various processes, including embryonic development, immunity, glucose metabolism, erythropoiesis, and angiogenesis [16]. Regulation of HIF mainly depends on oxygen tension, where, under normoxic conditions (usually physiologic conditions), HIF-1α hydroxylated by prolyl-hydroxylase domain-containing enzyme (PHD) is recognized and ubiquitylated by the von Hippel–Lindau (VHL) protein and ubiquitin-ligase complex. Then, ubiquitylated HIF-1α is degraded by the 26S proteasome. Conversely, under hypoxic or anoxic conditions, HIF-1α can escape from hydroxylation, and combined with the β subunit, the HIF complex can then bind to the promoters of HIF-responsive genes. Combined with several coactivators, such as p300/Creb-binding protein (CBP) and pyruvate kinase isoform M (PKM) 2, HIF initiates the transcription of genes involved in the abovementioned crucial biologic processes.

Because of rapid cell proliferation and aberrant angiogenesis, intratumoral hypoxia can easily occur during tumorigenesis and is associated with poor prognosis and treatment resistance [17]. Thus, the role of HIF-1α in growth, altered energy metabolism, invasion, and metastasis of cancer cells is important, and it may be an effective target for cancer treatment [18]. However, only limited numbers of studies have evaluated the effects of HIF-1α inhibitors on thyroid cancer progression [19,20].

IDF-11774, formerly known as LW6 [21], is an HIF-1α inhibitor that has shown effectiveness in several in vitro and in vivo models of colorectal [21] and lung cancer [22]. It promoted proteasomal degradation of HIF-1α to prevent accumulation by suppressing HIF-1α refolding and increasing intracellular oxygen tension [22]. Recently, IDF-11774 was found to suppress colon cancer cell metabolism [23]. However, the effect of IDF-11774 has not been evaluated in thyroid cancer cells.

In this study, we evaluated the status of HIF-1α expression in several thyroid cancer cell lines and the effects of HIF-1α inhibitor (IDF-11774) treatment on thyroid cancer progression.

## 2. Results

### 2.1. Expression of HIF-1α in Thyroid Cancer Cell Lines

**Figure 1:**
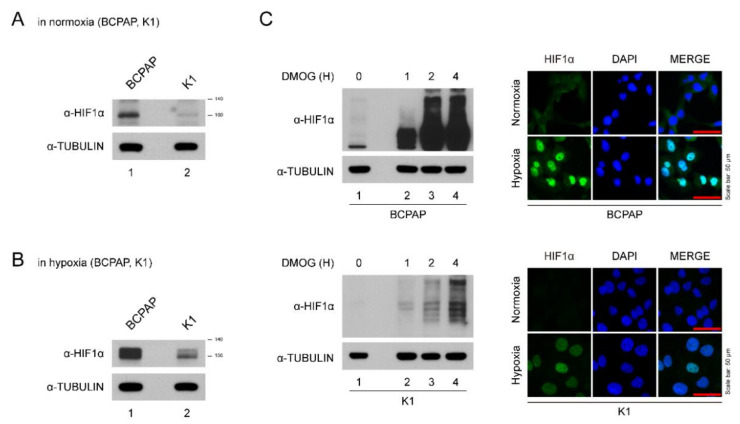
Expression of HIF-1α in thyroid cancer lines. (**A**) HIF-1α expression levels in different thyroid cancer cells under normoxia. Thyroid cancer cells (BCPAP and K1) were seeded and incubated in normoxic conditions. Cell lysates were immunoblotted with anti-HIF1α and anti-TUBULIN. (**B**) HIF-1α expression levels in different thyroid cancer cells under hypoxia (with DMOG for 4 h). Thyroid cancer cells (BCPAP and K1) were seeded and incubated in hypoxic conditions. Cell lysates were immunoblotted with anti-HIF1α and anti-TUBULIN. (**C**) Western blotting and immunofluorescence analysis of HIF-1α expression of BCPAP and K1 cells after DMOG treatment. (Left) Western blot analysis of HIF1α protein in hypoxia condition. BCPAP and K1 cells were seeded and treated with DMOG at indicated time point. Cell lysates were immunoblotted with anti-HIF1α and anti-TUBULIN. (Right) Immunofluorescence analysis of HIF1α protein under normoxia or hypoxia. BCPAP and K1 cells were stained with HIF1α antibody. Nuclei were counterstained with DAPI. Scale bar represents 50 μm (100×).

BCPAP cells expressed HIF-1α even under normoxic conditions (Figure 1A). Hypoxic insults (incubation in a hypoxia chamber and DMOG treatment) induced higher expression of HIF-1α levels in BCPAP cells (Figure 1B,C). The intensity of HIF-1α expression seen in K1 cells was much lower than in BCPAP cells (Figure 1A,B). For immunofluorescence staining, BCPAP cells were stained with HIF-1α antibody in both normoxic and hypoxic conditions in the nucleus while K1 was slightly stained (Figure 1C). Taken together, these data suggest that HIF-1α expression in BCPAP cells is highly induced and localized to the nucleus.

### 2.2. Expression of HIF-1α and Proliferation of BCPAP was Suppressed by the HIF-1α Inhibitor

**Figure 2:**
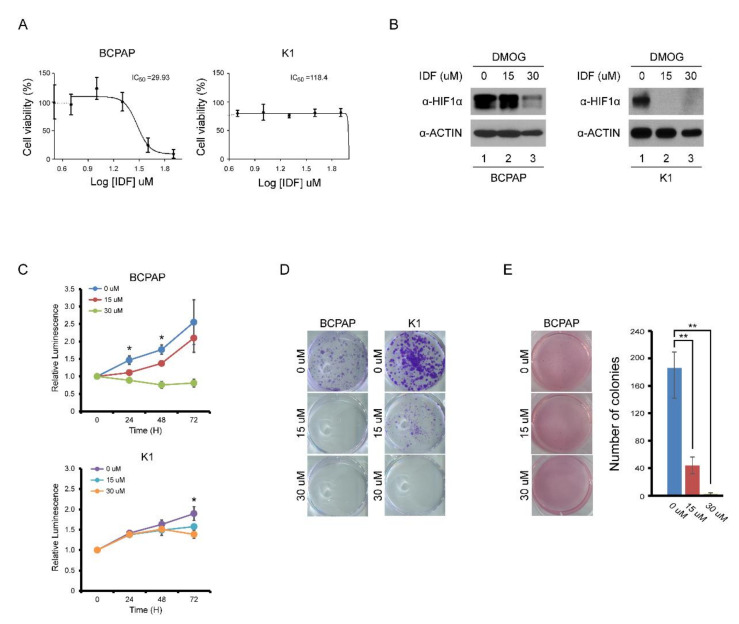
Effect of IDF-11774 on the proliferation of BCPAP and K1 cells. (**A**) IC_50_ analysis of IDF-11774I on BAPAP and K1 cells. (**B**) Western blotting analysis of HIF-1α expression in BCPAP and K1 cells after increasing doses of IDF-11774. After pretreatment with DMOG for 4 h, BCPAP and K1 cells were incubated with IDF-11774 for 24 h. Cell lysates were immunoblotted with anti-HIF1α and anti-ACTIN. (**C**) IDF-11774 treatment decreased cell viability of BCPAP cells in a dose-dependent manner but did not decrease the viability of K1 cells. Cell viability was measured at the indicated time using a CellTiter-Glo system. Data in the graph are presented as mean ± standard deviation of fold increase normalized by cells at day 0 (*n* = 5, * *p* < 0.01). (**D**) IDF-11774 treatment decreased colony formation of BCPAP cells in a dose-dependent manner but did not decrease colony formation in K1 cells. BCPAP and K1 cells were seeded and incubated with IDF for 1–2 weeks. Cell colonies were visualized by staining using crystal violet. (**E**) IDF-11774 treatment decreased anchorage-independent growth of BCPAP cells in a dose-dependent manner. BCPAP cells seeded and cultured for 3–4 weeks and cell colonies were visualized by staining with crystal violet. Data in the graph are presented as mean ± standard deviation (*n* = 3, ** *p* < 0.001).

The half maximal inhibitory concentration (IC_50_) for HIF-1α of IDF-11774 was determined at the dose of 30 μM (Figure 2A). After treatment of IDF-11774, the expression of HIF-1α in BCPAP and K1 cells decreased in a dose-dependent manner (Figure 2B). In cell viability and colony formation assays, the proliferation of BCPAP cells was inhibited by IDF-11774 in a dose-dependent manner (Figure 2C,D). In K1 cells, which did not show definite HIF-1α expression in normoxic conditions, the IC_50_ could not be calculated. No inhibitory effects on K1 cell proliferation were observed. In anchorage-independent growth, BCPAP cells dramatically decreased their colony number after IDF-11774 treatment in a dose-dependent manner (Figure 2E).

### 2.3. Migration and Invasion was Inhibited by the HIF-1α Inhibitor

**Figure 3:**
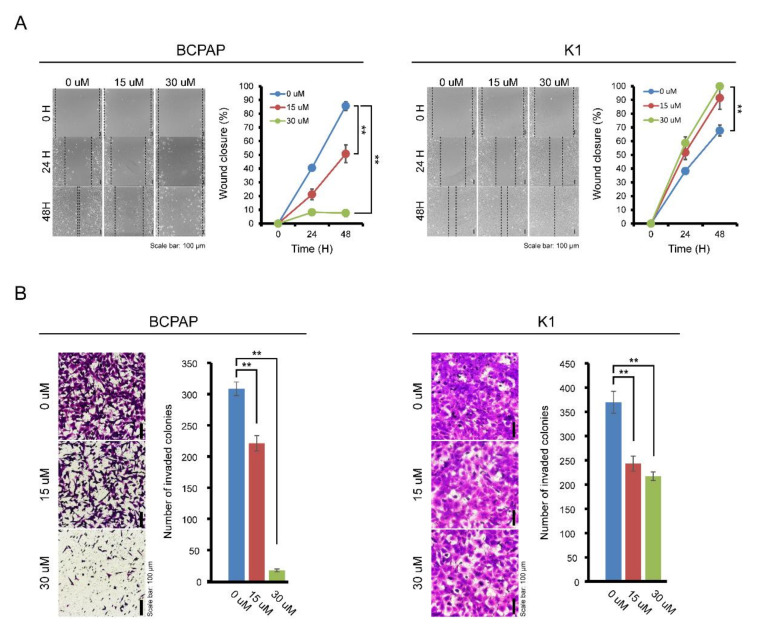
Effect of IDF-11774 on the migration and invasion properties of BCPAP and K1. (**A**) The effect of IDF-11774 on the migration of BCPAP and K1 cells was measured using a wound-healing assay. Data in the graph are presented as mean ± standard deviation of relative wound closure normalized by wound area at day 0 (*n* = 5, ** *p* < 0.001). (**B**) Effect of IDF-11774 on the cell invasion of BCPAP and K1 cells in a transwell invasion assay. Cell invasion was estimated, and data are presented as mean ± standard deviation of the number of invaded cells (*n* = 5, ** *p* < 0.001).

The effects of IDF-11774 on the migration and invasion of BCPAP cells were evaluated by wound healing and transwell invasion assays. As shown in Figure 3A, treatment with IDF-11774 effectively suppressed the migration of BCPAP in a dose-dependent manner. The maximum effect was observed after 48 h of IDF-11774 treatment. In K1 cells, treatment with IDF-11774 led to subtle suppression of migration. Invasiveness of BCPAP was highly attenuated by IDF-11774 treatment in the transwell invasion assay while that the invasiveness of K1 cells was slightly decreased (Figure 3B), suggesting that IDF-11774 reduced the migration and invasive abilities in BCPAP cells that showed prominent HIF-1α expression.

### 2.4. Cycloheximide Enhanced the Effect of IDF-11774 While Velcade Attenuated the Effect

**Figure 4:**
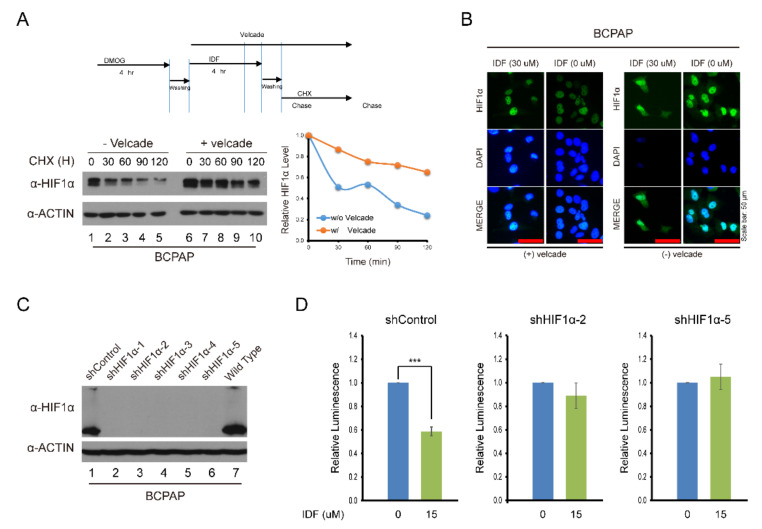
Alteration of the drug effect on HIF-1α expression after knockdown of HIF-1α and treatment with cycloheximide and velcade. (**A**) Analysis of HIF-1α expression after IDF-11774 treatment with cycloheximide and velcade. BCPAP cells were incubated with DMOG for 4 h. Cells were treated for 4 h with IDF-11774 alone or with IDF-11774 and velcade. The half-life of HIF1α was estimated by treating with cycloheximide at the indicated time. (**B**) Immunofluorescence of HIF-1α expression with IDF-11774 and velcade. BCPAP cells were treated with DMOG for 4 h and incubated for 24 h in the presence or absence of IDF-11774. Cells were stained with HIF1α antibody. Nuclei were counterstained with DAPI. Velcade treatment was administered for 5 h before immunostaining. Scale bar represents 50 μm (100×). (**C**) Analysis of HIF-1α expression in HIF-1α knockdown BCPAP cells. BCPAP cells stably expressing Control-shRNA or HIF1α-shRNAs were detected by Western blot analysis. (**D**) HIF-1α knockdown attenuated the effect of cell viability of BCPAP cells treated with IDF-11774. BCPAP Cells stably expressing shRNA were incubated with DMSO or IDF-11774. Cell viability was measured at the indicated time using the CellTiter-Glo system. Data in the graph are presented as mean ± standard deviation of fold increase normalized by cells at day 0 (*n* = 5, ** *p* < 0.001).

To explore the effect of IDF-11774 on HIF-1α stability and degradation, IDF-11774 combined with CHX, an inhibitor of de novo protein synthesis, decreased the expression of HIF-1α in BCPAP cells more efficiently than IDF-1174 without CHX. However, after co-treatment with velcade, a proteasome inhibitor, HIF-1α expression was not reduced (Figure 4A,B). In immunofluorescence staining, after IDF-11774 treatment, HIF-1α remained in the cytosol and induced cell death while IDF-11774 combined with velcade reduced the effects. These data indicate that IDF-11774 induced the death of BCPAP cells in a manner that is related to increased HIF-1α degradation.

### 2.5. Knockdown of HIF-1α by ShRNA

To investigate the effect of HIF-1α knockdown on tumor growth, we used a stable knockdown cell line produced by lentiviral transduction particles. The knockdown of HIF-1α by shRNA was observed as shown in Figure 4C. The effect of IDF-11774 was attenuated after knockdown of HIF-1α (Figure 4D).

## 3. Discussion

In the present study, expression of HIF-1α was seen in BCPAP cells but not K1 cells in normoxic and hypoxic conditions. The HIF-1α inhibitor IDF-11774 successfully inhibited the proliferation, migration, invasion, and anchorage-independent growth of BCPAP cells. However, expression of HIF-1α was absent in K1 cells under normoxic conditions, and proliferation and migration were not effectively inhibited by IDF-11774. Based on the HIF-1α expression changes after IDF-11774 treatment combined with an inhibitor of de novo protein synthesis (CHX) or a proteasome inhibitor (velcade), inhibition effects of IDF-11774 might result from post-transcriptional modulation, accelerating the degradation of HIF-1α. Knockdown of HIF-1α with shRNA attenuated the effects of IDF-11774 on BCPAP cell proliferation.

Various thyroid cancer cell lines have been developed and certified as being of thyroid origin [24]. Among them, the BCPAP cell line, which contains mutant BRAF, has been widely studied. In our study, expression of HIF-1α in BCPAP cells, even under normoxic conditions, was re-identified as some of previous studies’ BCPAP [19,25,26]. Notably, HIF-1α was detected even without hypoxic stimuli, which implies that the BCPAP cell line is a good model for research of thyroid cancer where HIF-1α is highly expressed. Previous study showed different genome alterations other than BRAF between K1 and BCPAP cells, thus it could be intriguing to evaluate the role of gene involved in histone modification (such as SETD2 and CREBBP) under different hypoxic stress [27]. In addition, as manipulation of the BRAF mutation was correlated in HIF-1α expression in vitro studies of melanoma cell lines [28] and the thyroid cancer cell line (BCPAP) [29], the link between BRAF mutation and Hif-1α expression is highly suspected. Thus, evaluation of the effects of HIF-1α inhibition in the mutant BRAF thyroid cancer cell line (BCPAP) would have a significant clinical implication in terms of the high BRAF mutation prevalence in thyroid cancer and its association with poor outcomes.

The role of HIF-1α in cancer development has been widely investigated as it is a key molecule for cellular adaptation in response to hypoxia, which is commonly encountered during tumorigenesis. In various types of cancers, such as melanoma, breast cancer, and colorectal cancer, HIF-1α has been associated with aggressiveness and poor outcomes [17]. In addition, high expression of HIF-1α is related to resistance to chemotherapy [30] and radiation therapy [31]. Therefore, it has been an attractive target for the treatment of advanced cancers. Several HIF-1α inhibitors, which have different inhibitory mechanisms, including transcriptional and post-transcriptional modulations, have demonstrated successful suppression of cancer cell growth [32].

The xpression of HIF-1α in human thyroid cancer tissues is associated with tumor aggressiveness [14,15], and dedifferentiated anaplastic thyroid cancer has shown particularly high expression of HIF-1α [25]. In one study that evaluated the expression of HIF-2α in papillary thyroid cancer tissue, HIF-2α was associated with a high prevalence of lymph node metastasis [33]. Interestingly, a link between HIF-1α and BRAF mutations, which are a prevalent and well-known poor prognostic factor in papillary thyroid cancer [34], was suggested in thyroid cancer [29]. It has also been shown in follicular thyroid cancer cell lines that epithelial–mesenchymal transition is induced by HIF-1α activation [35]. Like other cancers, HIF-1α is likely to play a pivotal role in the progression of thyroid cancer.

However, few studies have investigated HIF-1α as a target for thyroid cancer treatment. 17-N-allylamino-17-demethoxygeldanamycin (AAG) effectively promoted HIF-1α degradation in an orthotopic mouse model [19]. In follicular thyroid cancer cell lines, GDC-0941, a PI3K inhibitor that sequentially inhibits HIF-1α pathways, has been found to attenuate metastatic behavior [20]. Consistent with the prior two studies, our results also showed that inhibition of HIF-1α by IDF-11774 had effects on cancer progression. IDF-11774, which is similar to 17-AAG, modulates HIF-1α at post-translational levels [21,22,23]. Of note, in TCGA data, alterations in mRNA expression of HIF-1α were not prominent (data not shown). Thus, HIF-1α could be controlled by post-transcriptional modulation, and drugs that modulate HIF-1α stability at post-transcriptional levels might be effective treatments in thyroid cancer. In this context, HIF-1α inhibitors, which target post-transcriptional processes, could be an appropriate treatment for thyroid cancer. We found that IDF-11774 effectively suppressed HIF-1α by accelerating its degradation, and successful suppression of HIF-1α resulted in the attenuation of tumor progression in terms of proliferation, migration, and invasion.

## 4. Materials and Methods

### 4.1. Cell Culture

BCPAP and K1 cells were obtained from ATCC (Manassas, VA, USA) and Sigma-Aldrich (Saint Louis, MO, USA) respectively. The papillary thyroid carcinoma cell line BCPAP (derived from a poorly differentiated PTC) was cultured in RPMI 1640 medium (Hyclone, Salt Lake City, UT, USA) supplemented with 10% fetal bovine serum. The K1 (derived form a metastasis of a well-differentiated PTC) cell line was cultured in DMEM: Ham’s F12 (Hyclone): MCDB 105 (Sigma) (2:1:1) medium containing 2 mM Glutamine and 10% fetal bovine serum.

For hypoxic conditions, cells were grown in a hypoxia chamber (Anaerobic System by Thermo Fisher Scientific (Waltham, MA, USA) <0.1% O_2_) [20,25] or in medium containing 100 μg/mL DMOG (dimethyloxalylglycine, Sigma-Aldrich) [36,37]. Through competitive inhibition of PHD, DMOG could stabilize HIF-1α and is widely utilized to mimic hypoxic conditions [36,38].

### 4.2. RNA Interference

Lentiviral transduction particles for the stable HIF-1α knockdown were purchased from Sigma-Aldrich. Cells were seeded at a density of 10^5^ cells/well in a 6-well plate. Infected cells were selected for at least three days in 2 μg/mL puromycin. Lentiviral transduction particles included TRCN0000003806, TRCN0000003809, TRCN0000003810, TRCN0000003811, and TRCN0000000819.

### 4.3. Western Blotting for HIF-1α

Cells were lysed in lysis buffer (40 mM Tris-Cl pH 7.5, 150 mM NaCl, 1% SDS) and the protein extract was used for analysis by Western blotting. Antibodies included anti-HIF-1α (1:1000, 610958, BD biosciences), anti-β-ACTIN (1:10,000, sc-47778, Santa Cruz Biotechnology, Dallas, TX, USA), and anti-α-TUBULIN (1:10,000, ab7291, Abcam). β-ACTIN and α-TUBULIN were used as loading control. To determine the proteasome degradation and stability of HIF-1α, cells were cultured in medium containing cycloheximide (CHX, 50 μg/mL) with or without velcade (bortezomib, 100 μg/mL). After, cells were harvested at indicated time points and analyzed by Western blotting using HIF-1α antibodies.

### 4.4. Cell Viability Assay

Proliferation was evaluated using the CellTiter-Glo Luminescent Cell Viability Assay Kit (Promega, Madison, WI, USA) and the colony formation assay. To determine the cell viability using the CellTiter-Glo assay kit, BCPAP and K1 cells were seeded 2 × 10^3^ cells/well with DMSO, 15 μM IDF-11774, or 30 μM IDF-11774 per well. Cells were analyzed on days 1–3. At the end of the incubation period, plate and CellTiter-Glo reagent were equilibrated at room temperature for 30 min. Then, 100 μL of reagent were added and mixed for 3 min on an orbital shaker. Subsequently, the plate was incubated at room temperature for 10 min. Luminescence intensity was determined using a GloMax*^®^* 96 Microplate Luminometer (Promega).

To determine the cell proliferation with the colony forming assay, BCPAP and K1 cells were seeded at 10^3^ cells/well with DMSO, 15 μM IDF-11774, or 30 μM IDF-11774 in 6-well dishes. Cells were cultured in medium for 1–2 weeks. The cells were then fixed with 3.7% paraformaldehyde/sucrose and stained with 0.5% crystal violet for 30 min.

### 4.5. Soft Agar Assay

To estimate anchorage-independent growth, BCPAP cells were seeded at 2 × 10^3^ cells in 0.35% top agar in medium over a layer of 0.7% base agar in a 6-well plate with DMSO, 15 μM IDF-11774, or 30 μM IDF-11774. The plates were incubated at 37 °C for 3–4 weeks. The experiment was performed in triplicate. The cell colonies were visualized by staining with 0.004% crystal violet/2% ethanol in PBS. Cells were counted under light microscopy.

### 4.6. Wound Healing Assay

In the wound healing assay, wound closure was observed at indicated incubation times with DMSO, IDF 15 μM IDF-11774, or 30 μM IDF-11774. BCPAP and K1 cells were seeded at 5 × 10^5^ cells per well in 6-well plates and incubated. Cell monolayers were wounded with a sterile 200-μL pipette tip and washed with medium to remove detached cells and fresh medium with DMSO, 15 μM IDF-11774, or 30 μM IDF-11774 was added. Cell migration was evaluated by measuring the differences in the areas of the wounds.

### 4.7. Transwell Invasion Assay

Cells were seeded at 10^5^ (BCPAP) or 3 × 10^4^ (K1) cells with DMSO, 15 μM IDF-11774, or 30 μM IDF-11774 per well in the upper chamber of a transwell insert (8-μm polycarbonate membrane; iNtron, Bethesda, MD, USA) coated with 1–2 mg of Matrigel (BD Biosciences, San Jose, CA, USA) that was placed in a 24-well plate. The lower chamber was filled with 800 μL of medium supplemented with 10% fetal bovine serum and incubated for 16–24 h. Cells that invaded through the Matrigel to the underside of the membrane were fixed with 3.7% paraformaldehyde/sucrose and stained with 0.5% crystal violet for 1 min. Cells that remained at the upper surface of the insert were removed with a cotton swab, and the invaded cells were counted in five random fields under light microscopy.

### 4.8. Immunofluorescence

Cells grown on coverslips were rinsed in PBS and fixed in 3.7% (*w/v*) paraformaldehyde/sucrose. Cells were washed with PBS and permeabilized with 0.4% TritonX-100 in PBS for 20 min. Coverslips were blocked for 1 h and incubated for 1 h at room temperature with HIF-1α antibody and then washed 3 times for 5 min in PBS and incubated for 1 h with the Alexa Fluor Goat anti-mouse 488 (Invitrogen, Carlsbad, CA, USA). After washes in PBS, coverslips were dipped in water and then dried. The coverslips were embedded in ProLong Gold (Invitrogen) supplemented with 0.1 μg/mL DAPI and imaged with fluorescence microscopy.

### 4.9. Statistical Analysis

All data were analyzed using SPSS 16.0 (SPSS Inc., Chicago, IL, USA). The results are presented as the mean ± standard deviation of at least three independent experiments. Comparisons between two groups were performed using Student’s *t*-tests. A *p*-value of <0.05 was considered to be statistically significant.

### 4.10. Drug Treatment

IDF-11774 (provided by Ildong Pharmaceutical Co., Ltd., Seoul, Korea) was dissolved in DMSO at 10 mM and the stock solution was stored at −20 °C.

## 5. Conclusions

Among thyroid cancer cell lines, BCPAP cells expressed HIF-1α in both normoxic and hypoxic conditions. Treatment with the HIF-1α inhibitor IDF-11774 successfully suppressed the progression of BCPAP by enhancing the degradation of HIF-1α. HIF-1α inhibition could be a therapeutic target for thyroid cancers with high HIF-1α expression.
